# 25-hydroxyvitamin D, influenza vaccine response and healthcare encounters among a young adult population

**DOI:** 10.1371/journal.pone.0192479

**Published:** 2018-02-09

**Authors:** Rachel U. Lee, Seung Hyun Won, Christian Hansen, Nancy F. Crum-Cianflone

**Affiliations:** 1 Division of Allergy and Immunology, Department of Internal Medicine, Naval Medical Center San Diego, San Diego, CA; 2 Infectious Disease Clinical Research Program, Department of Preventive Medicine and Biostatistics, Uniformed Services University of the Health Sciences, Bethesda, MD; 3 Henry M. Jackson Foundation for the Advancement of Military Medicine, Bethesda, MD; 4 Operational Infectious Disease Department, Naval Health Research Center, San Diego, CA; 5 Division of Infectious Disease, Department of Internal Medicine, Scripps Mercy Hospital, San Diego, CA; University of North Carolina at Chapel Hill, UNITED STATES

## Abstract

**Background:**

Influenza causes significant morbidity and mortality; the pandemic in 2009–2010 was a reminder of the potential for novel strains and antigenic changes. Studies have shown that vitamin D deficiency may be associated with poor vaccine immunogenicity, therefore we sought to determine if there was a correlation between 25-hydroxyvitamin D [25(OH)D] and influenza vaccine response.

**Methods:**

A retrospective observational study was conducted among young, healthy military members to evaluate the association between total 25(OH)D levels with post influenza vaccination antibody titers and healthcare encounters during the 2009–10 influenza season. Univariate analyses were performed to evaluate whether 25(OH)D levels are associated with baseline characteristics and post-vaccination antibody responses. Multivariable logistic regression models were utilized to determine the associations between antibody responses and 25(OH)D levels adjusting for possible confounders.

**Results:**

A total of 437 subjects were studied. Most participants were young adults (91% were 18–39 years of age), 50% were male, and 56% resided in the southern U.S. Overall, 152 (34.8%) were vitamin D deficient, 167 (38.2%) insufficient, and 118 (27.0%) with normal 25(OH)D levels. There were no demographic differences by 25(OH)D category. Only 224 (51.3%) demonstrated a seroprotective anti-influenza post-vaccination titer, which did not vary by categorical 25(OH)D levels [vitamin D deficient vs. normal: OR 1.10 (0.68–1.78) and insufficient vs. normal: OR 1.25 (0.78–2.01)] or continuous vitamin D levels [OR 0.98 (0.84–1.15)]. There were also no associations with increased influenza like illnesses, respiratory diagnoses and healthcare encounters between the vitamin D groups.

**Conclusion:**

Vitamin D insufficiency and deficiency were highly prevalent despite evaluating a young, healthy adult population. There were no significant associations between 25(OH)D levels and post-vaccination antibody titers to influenza vaccine. Further studies are required to discover strategies to improve vaccine efficacy as well as to determine the role of 25(OH)D in vaccine immunity.

## Introduction

Influenza causes seasonal epidemics resulting in serious illness and death worldwide. In the U.S., the annual burden of influenza is estimated to be 25 to 50 million cases, resulting in an average of 225,000 hospitalizations and up to 48,000 related deaths [1–2]. In April 2009, a novel H1N1 swine-origin influenza A (pH1N1) was identified as the source of a worldwide pandemic, resulting in morbidity and mortality, particularly in children and young adults [3–5]. Despite public awareness of influenza and widespread availability of vaccinations, there has been little effect on reducing the morbidity and mortality associated with influenza over the years. While this is partly driven by low vaccine uptake, it is also related to suboptimal post-vaccination immune responses [6–8]. In recent studies, efficacy of seasonal influenza vaccine ranged from 16% to 88% depending on the population, type of vaccine, match between the circulating and vaccine strains, and host factors [9–11].

Vitamin D deficiency is widely recognized in all populations [12–13]. Low 25-hydroxyvitamin D [25(OH)D] levels have been associated with a variety of disease states, including susceptibility to infections [14–19]. Meta-analyses have shown that vitamin D has a protective effect against respiratory tract infections, and data supports the inverse relationship of 25(OH)D levels and the incidence of influenza. [20–22]. Vitamin D receptors are present on many immune cells that induce genes encoding antimicrobial peptides active against influenza and other pathogens [23–24]. Also, evidence suggests that vitamin D has immunomodulatory functions which influence immune pathways by boosting innate immunity while modulating excessive inflammation [25–26].

Studies have shown that vitamin D supplementation enhanced response to various vaccinations [27–28]. Further, vitamin D insufficiency has been associated with poorer responses to the human papillomavirus (HPV), hepatitis B virus, and influenza vaccines in specific populations [29–31]. One study examined the effect of vitamin D supplementation on tetanus toxoid booster vaccination and found improved tetanus toxoid specific IgG with vitamin D, while another study evaluating the administration of calcitriol with influenza vaccine did not demonstrate benefit [32–33]. Other studies that have shown no demonstrable difference in vaccine responses with 25(OH)D levels or supplementation; however, most of these studies have been small and were performed in select populations with varied methodologies [33–37].

The recent novel H1N1 pandemic has demonstrated that more studies are needed to adequately prepare for such future pathogens. In particular, host immunity and response to vaccination require further study. Therefore, we sought to investigate the associations of 25(OH)D levels on post-vaccination influenza serology and relevant healthcare outcomes in a young adult population.

## Materials and methods

### Participants and study design

A retrospective cross-sectional observational study was performed to evaluate post-vaccination antibody titers and clinical outcomes after vaccination with the 2009 monovalent influenza A (H1N1) vaccine (strain A/California/7/2009/H1N1) in 440 adult subjects and their associated 25(OH)D levels. The 2009 pH1N1 vaccine was chosen specifically because there was no recent vaccination or circulation of the 2009 pH1N1 strain prior to the onset of the pandemic, and it was the predominant circulating influenza virus during the 2009–2010 season. Additionally, the monovalent pH1N1 vaccination was well matched to the predominant circulating influenza strain [38].

The study population and serum were drawn from the Armed Forces Health Surveillance Center (AFHS) and the Defense Medical Surveillance System (DMSS), which are large databases containing longitudinal data including demographics, immunizations, and medical encounters as well as stored blood specimens for US military service members [39]. The serum repository maintains serum at -30 degree Celsius and as the largest known bank of human sera; as such, it has supported many public health investigations and research [40]. Inclusion criteria for the current study included service members age ≥18 years located in the continental United States and who received the first monovalent pH1N1 vaccine during the 2009–2010 influenza season and had a serum sample at least 30 days post-vaccination in February of 2010. A single month was chosen to minimize the temporal variation of 25(OH)D levels. In order to be able to reflect the general U.S. population, inclusion of females in 50% of the samples were requested. The study protocol was approved by the Naval Health Research Center Institutional Review Board in compliance with all applicable Federal regulations governing the protection of human subjects; consent was waived as all data was collected retrospectively and de-identified.

The primary study outcome was to determine whether there was an association between influenza vaccine immunogenicity and serum 25(OH)D levels. We also evaluated healthcare encounters during the risk period as well as those specifically resulting in a primary diagnosis consistent with an influenza-like illness (ILI) using codes from the International Classification of Disease, Ninth Revision, Clinical Modification (ICD-9-CM). Subject data collected included age, sex, rank, and geographic location. Geographic regions were defined by the Census Bureau and based on the 4 geographic regions; due to locations of military bases, not all areas may have been equally represented.

### Laboratory testing

Influenza antibody titers to the monovalent 2009 influenza A (H1N1) vaccine (strain A/California/7/2009(H1N1)) were determined using the microneutralization (MN) test conducted using standardized reagents in the World Health Organization (WHO) Influenza Reagent Kit as previously described [41]. Seroprotection was defined as a titer of ≥1:40 post-vaccination. Serum levels of 25(OH)D were determined using commercially available kits (Eagle Biosciences, Nashua, NH, USA) according to the manufacturer’s instructions. This assay measure total 25(OH)D, which includes both 25(OH)D2 and 25(OH)D3. Samples were batched and tested at the same time at the Naval Health Research Center laboratory. External quality validation was obtained and performed with five samples from the Vitamin D External Quality Assessment Scheme (DEQAS), a program that monitors performance and accredits testing of serum 25(OH)D [42].

### Clinical outcome measures

Healthcare utilization records were evaluated up to 365-days post vaccination using DMSS. Electronic medical records of inpatient and outpatient healthcare encounters of each subject were used to compare the frequency of relevant healthcare encounters by ICD-9 codes (endocrine/immune, ICD 240–279; respiratory diagnoses, ICD 460–519; diseases of skin, 680–709; general syndromes, 780–799). Specific ILIs were evaluated using previously defined ICD-9 codes used in prior studies corresponding to laboratory diagnosed influenza (079.99, 382.9, 460, 461.9, 465.8, 465.9, 466, 486, 487, 487.1, 487.8, 490, 780.6 and 786.2) [43]. Although laboratory-confirmed influenza outcomes would be more specific, most cases of ILI do not undergo influenza testing. Subjects were examined for ILI starting 14 days after vaccination until 365 days later and censored after the first diagnosis.

### Statistical analysis

Descriptive statistics were reported as medians (interquartile range) and frequencies (percentage) for continuous and categorical variables, respectively. 25(OH)D levels were evaluated as categorized levels with three groups: normal (>30 ng/ml), insufficient (20–30 ng/ml), and deficient (<20 ng/ml). The baseline characteristics, antibody responses, and distributions of healthcare outcomes among the vitamin D groups were compared using Chi-square or Fisher’s exact test and Kruskal-Wallis test as appropriate. Odds ratios were calculated by logistic regression models to evaluate the associations between serum 25(OH)D levels and the outcome of seroprotection post-vaccination (seroprotective titer < or ≥ 1:40) with or without adjusting for possible confounders. All of the statistical tests were two-sided with statistical significance defined at p-value < 0.05 and performed with SAS version 9.4 (SAS Institute Inc, Cary, NC).

## Results

### Study population characteristics

440 subjects were included initially, however 3 were excluded due to insufficient serum for laboratory testing. Baseline population characteristics of the 437 subjects are presented in Table 1. The population consisted of young adults, with 396 (90.6%) in the 18–39 years age range. There was a similar distribution of males and females (50.3% vs. 49.7%; p = 0.82). The majority of participants resided in the South (55.8%), followed by the West (29.3%), then Midwest (9.6%), and Northeast (5.3%). Regarding military status, 360 (82.4%) were enlisted service members. Influenza vaccination was given in the fall of 2009 in 334 (76.4%), and 370 (84.7%) received the injectable regular pH1N1 vaccine, whereas a smaller proportion received the vaccine in the winter or the preservative free or nasal formulation (Table 1).

**Table 1:** Baseline characteristics by serum 25(OH)D levels.

		25(OH)D level			
Characteristics, N(%)	Total	Normal	Insufficient	Deficient	P-value*
**Number of Subjects**	437	118	167	152	
		(27.0)	(38.2)	(34.8)	
**Gender**					
Male	220	57	87	76	0.82
	(50.3)	(48.3)	(52.1)	(50.0)	
Female	217	61	80	76	
	(49.7)	(51.7)	(47.9)	(50.0)	
**Age (years)**					
18–24	185	43	74	68	0.66
	(42.3)	(36.4)	(44.3)	(44.7)	
25–39	211	63	77	71	
	(48.3)	(53.4)	(46.1)	(46.7)	
40+	41	12	16	13	
	(9.4)	(10.2)	(9.6)	(8.6)	
**Service Rank**					
Enlisted	360	100	134	126	0.60
	(82.4)	(84.7)	(80.2)	(82.9)	
Officer/Warrant	77	18	33	26	
	(17.6)	(15.3)	(19.8)	(17.1)	
**Vaccine Type**					
Novel H1N1-09 NASAL	31	12	10	9	0.70
	(7.1)	(10.2)	(6.0)	(5.9)	
Novel H1N1-09 PRE. FREE	28	10	10	8	
	(6.4)	(8.5)	(6.0)	(5.3)	
H1N1-09 INJ	370	94	144	132	
	(84.7)	(79.7)	(86.2)	(86.8)	
Novel H1N1-09 NASAL PRE. FREE	8	2	3	3	
	(1.8)	(1.7)	(1.8)	(2.0)	
**Year of Vaccine**					
2009	334	90	132	112	0.53
	(76.4)	(76.3)	(79.0)	(73.7)	
2010	103	28	35	40	
	(23.6)	(23.7)	(21.0)	(26.3)	
**Service Location**					
Northeast	23	6	9	8	0.93
	(5.3)	(5.1)	(5.4)	(5.3)	
Midwest	42	10	17	15	
	(9.6)	(8.5)	(10.2)	(9.9)	
South	244	72	91	81	
	(55.8)	(61.0)	(54.5)	(53.3)	
West	128	30	50	48	
	(29.3)	(25.4)	(29.9)	(31.6)	
**Time from Vaccination to Serum Collection (days)**	64.0	63.5	65.0	63.0	0.80
	(38.0)	(41.0)	(37.0)	(37.0)	

Vitamin D level: Normal (>30ng/ml), insufficient (20-30ng/ml), and deficient (<20ng/ml).

Frequency (%) and Median (IQR) for categorical and continuous variables, respectively.

*Chi-square or Fisher’s exact and Kruskal-Wallis tests for categorical and continuous variables, respectively.

### 25(OH)D levels

Overall, 152 (34.8%) participants were vitamin D deficient (<20 ng/ml), 167 (38.2%) were insufficient (20–30 ng/ml), and 118 (27.0%) had normal (> 30ng/ml) 25(OH)D levels. There were no statistically significant differences in baseline characteristics among the vitamin D groups (Table 1).

### Study outcomes

The associations between antibody GMT responses and 25(OH)D levels are summarized in Table 1. Overall, 51% of vaccinees developed seroprotection defined as a titer of ≥1:40 post-vaccination. The development of a seroprotective response or the median GMTs did not significantly differed by the 25(OH)D categories (Table 2).

**Table 2:** Outcomes of GMT titers and medical diagnoses by serum 25(OH)D levels.

		25(OH)D level			
Characteristics	Total	Normal	Insufficient	Deficient	P-value*
**Number of Subjects**	437	118	167	152	
		(27.0)	(38.2)	(34.8)	
**Geometric Mean Titer (GMT)**					
GMT	40.0	24.1	40.0	40.0	0.51
	(70.0)	(75.0)	(70.0)	(70.0)	
< 40	213	61	77	75	0.64
	(48.7)	(51.7)	(46.1)	(49.3)	
≥ 40	224	57	90	77	
	(51.3)	(48.3)	(53.9)	(50.7)	
**Healthcare Visit**					
Never	140	30	59	51	0.19
	(32.0)	(25.4)	(35.3)	(33.6)	
At least once	297	88	108	101	
	(68.0)	(74.6)	(64.7)	(66.5)	
**Number of Medical Diagnosis**					
None	140	30	59	51	0.26
	(32.0)	(25.4)	(35.3)	(33.6)	
1	131	44	46	41	
	(30.0)	(37.3)	(27.5)	(27.0)	
≥ 2	166	44	62	60	
	(38.0)	(37.3)	(37.1)	(39.5)	
**Respiratory Diagnoses**					
No	330	89	125	116	0.95
	(75.5)	(75.4)	(74.9)	(76.3)	
Yes	107	29	42	36	
	(24.5)	(24.6)	(25.1)	(23.7)	
**Influenza-like Illness (ILI)**					
No	344	98	128	118	0.40
	(78.7)	(83.1)	(76.6)	(77.6)	
Yes	93	20	39	34	
	(21.3)	(16.9)	(23.4)	(22.4)	
**Endocrine/Immune Diagnosis**					
No	375	96	147	132	0.26
	(85.8)	(81.4)	(88.0)	(86.8)	
Yes	62	22	20	20	
	(14.2)	(18.6)	(12.0)	(13.2)	
**Syndrome**					
No	212	50	84	78	0.29
	(48.5)	(42.4)	(50.3)	(51.3)	
Yes	225	68	83	74	
	(51.5)	(57.6)	(49.7)	(48.7)	
**Diagnosis of the Skin**					
No	365	101	141	123	0.54
	(83.5)	(85.6)	(84.4)	(80.9)	
Yes	72	17	26	29	
	(16.5)	(14.4)	(15.6)	(19.1)	

Vitamin D level: Normal (>30ng/ml), insufficient (20-30ng/ml), and deficient (<20ng/ml).

Frequency (%) and Median (IQR) for categorical and continuous variables, respectively.

*Chi-square or Fisher’s exact and Kruskal-Wallis tests for categorical and continuous variables, respectively.

All medical diagnoses were grouped by the 25(OH)D status of normal, insufficient or deficient and reported in Table 2. Among the 437 subjects, 297 (68.0%) subjects had a healthcare visit at least once and diagnosed with one of these medical diagnoses: respiratory dx, endo/immune dx, syndrome, skin dx and ILI in the following year Overall, 107 (24.5%) subjects had a respiratory diagnosis and 93(21.3%) subjects had an ILI during the study period(Table 2).

Factors associated with antibody response (seroconversion) are shown in Table 3. There were no significant associations between seroprotection and 25(OH)D levels: vitamin D deficient vs. normal [OR 1.10 (0.68–1.78)] and insufficient vs. normal [OR 1.25 (0.78–2.01)]. Analyzing the 25(OH)D levels as continuous variables also demonstrated no significant association [OR 0.98 (0.84–1.15)]. Antibody response was not associated with any baseline characteristics (p-value >0.05) (Table 3). Multivariable logistic regression adjusted for the baseline characteristics shown in Table 4 did not detect any significant associations between antibody response and 25(OH)D levels (either as a categorical or continuous variable). Finally, we examined the associations between antibody responses (GMT as a continuous variable) and 25(OH)D levels as categorical variables and then as a continuous variables and found no associations in Table 4 (p-values 0.51 and 0.50, respectively).

**Table 3:** Factors associated with a seroprotective antibody responses (GMT ≥40 vs. <40).

	Unadjusted OR*	P-value
**25(OH)D Level**		
Insufficient vs. Normal	1.25 (0.78–2.01)	0.64
Deficient vs. Normal	1.10 (0.68–1.78)	
25(OH)D Level (by 10 ng/ml)	0.98 (0.84–1.15)	0.83
**Gender**		
Female vs. Male	0.86 (0.59–1.25)	0.42
**Age**		
25–39 vs. 18–24 years	0.75 (0.50–1.11)	0.28
40+ vs. 18–24 years	0.69 (0.35–1.36)	
**Service Rank**		
Officer/Warrant vs. Enlisted	0.66 (0.40–1.09)	0.11
**Healthcare Visit**		
At least once vs. Never	0.84 (0.56–1.25)	0.39
**Number of Medical Diagnoses**		
1 vs. None	0.91 (0.56–1.47)	0.56
2 or more vs. None	0.78 (0.50–1.23)	
**Medical Diagnosis (Yes vs. No)**		
Respiratory Diagnosis	0.75 (0.48–1.16)	0.19
Influenza-like Illness (ILI)	0.86 (0.55–1.37)	0.53
Endocrine/Immune Diagnosis	0.94 (0.55–1.61)	0.83
Syndrome	0.92 (0.63–1.34)	0.66
Diagnosis of the Skin	1.01 (0.61–1.67)	0.98
**Year of Vaccine**		
2010 vs. 2009	1.18 (0.76–1.83)	0.47
**Service Location**		
Northeast vs. South	1.28 (0.54–3.03)	0.16
Midwest vs. South	1.97 (0.99–3.92)	
West vs. South	0.87 (0.57–1.33)	
**Time from Vaccination to Serum Collection (days)**	1.00 (0.99–1.01)	0.43

*Unadjusted OR with its 95% CI and p-value from a simple logistic regression with GMT ≥ 40 as the event

**Table 4:** Association between clinical outcomes and vitamin D level.

	Unadjusted OR	P-value	Adjusted OR*	P-value
**Geometric Mean Titer (≥ vs. <40)**				
Insufficient vs. Normal	1.25 (0.78–2.01)	0.64	1.22 (0.76–1.96)	0.69
Deficient vs. Normal	1.10 (0.68–1.78)		1.07 (0.66–1.73)	
**Healthcare Visit (At least once vs. Never)**				
Insufficient vs. Normal	0.62 (0.37–1.05)	0.19	0.64 (0.37–1.10)	0.25
Deficient vs. Normal	0.68 (0.40–1.15)		0.68 (0.39–1.21)	
**Respiratory Diagnosis (Yes vs. No)**				
Insufficient vs. Normal	1.03 (0.60–1.78)	0.95	1.08 (0.61–1.91)	0.91
Deficient vs. Normal	0.95 (0.54–1.67)		0.96 (0.54–1.73)	
**Influenza like Illness Diagnosis (Yes vs. No)**				
Insufficient vs. Normal	1.49 (0.82–2.72)	0.40	1.55 (0.84–2.86)	0.35
Deficient vs. Normal	1.41 (0.76–2.61)		1.43 (0.77–2.67)	
**Endocrine/Immune Diagnosis (Yes vs. No)**				
Insufficient vs. Normal	0.59 (0.31–1.15)	0.26	0.64 (0.32–1.27)	0.41
Deficient vs. Normal	0.66 (0.34–1.28)		0.72 (0.36–1.43)	
**Syndrome (Yes vs. No)**				
Insufficient vs. Normal	0.73 (0.45–1.17)	0.29	0.75 (0.45–1.25)	0.37
Deficient vs. Normal	0.70 (0.43–1.13)		0.70 (0.42–1.17)	
**Diagnosis of the skin (Yes vs. No)**				
Insufficient vs. Normal	1.10 (0.57–2.13)	0.55	1.15 (0.59–2.24)	0.50
Deficient vs. Normal	1.40 (0.73–2.70)		1.46 (0.75–2.83)	

* ORs with its 95% CI and p-values from a logistic regression; adjusted for age, gender, time from vaccination to serum collection

## Discussion

High rates of vitamin D insufficiency and deficiency have been recognized worldwide, including in our healthy, young adult military population, where 73% had insufficient or deficient levels. Also, despite vaccination of a healthy, young adult population, only half (51%) developed seroprotective titers. Given that influenza vaccine effectiveness is often suboptimal, identifying modifiable host factors to improve clinical protection is crucial. We evaluated if low 25(OH) D levels would explain poor vaccination antibody responses, however, we found no relationship between 25(OH) D levels and seroprotective response to pH1N1 vaccination. Further, we found no associations with healthcare encounters for ILI or respiratory diagnoses.

Despite the variety of innate and adaptive immune functions that vitamin D may impact [12,15], studies have been conflicting thus far, with some studies showing a beneficial role of vitamin D and vaccination responses, while others have shown no effect [27–36]. Although treatment levels of 25(OH)D (≥20ng/ml) are well established for skeletal health (and prevention of rickets), levels for optimal health and primary prevention levels are not well established. We used the current clinical cut-offs for insufficiency (20–30 ng/ml) and deficiency (<20ng/ml), however some experts have shown that higher levels (≥40ng/ml) may be required [44].

Most studies measure antibody response as a surrogate of host protection against the pathogen and response to the vaccine; innate function and cellular immunity are less readily measurable yet studies show that vitamin D’s effects on innate immunity influence adaptive responses [28, 45–46]. Several studies have shown a correlation between 25(OH)D and enhanced vaccine response to tetanus, hepatitis B, and HPV [29–30, 32]. There have also been studies looking specifically at influenza vaccine due to the seasonal nature and variations in vitamin D; they have shown mixed results and vary widely in populations tested and methodologies. Chadha et al demonstrated a significant effect of 25(OH)D on serologic response to the influenza vaccine in a group of patients with prostate cancer [31], while Kriesel et al and Principi et al showed no benefit of calcitriol or vitamin D supplementation on influenza vaccine response in young healthy adults or children [33,35]. Other studies have since shown that there is no significant correlation between 25(OH)D levels and influenza vaccine response, as measured by antibody response [34–37]. Our results were consistent with the latter findings as there were no significant associations between antibody titers and 25(OH)D levels in our population.

A recent study demonstrated that vitamin D deficiency alone did not have significant detriment to influenza vaccine response, however combined vitamin A and D deficiencies reduced antibody responses in the respiratory tract and supplementation of both restored the specific antibody response [47]. Hence, vitamin D may be only one part of the equation to improving vaccine efficacy and other micronutrients and factors may require synergy for optimal efficacy. As such, additional research on the role of vitamin D in combination with other modifiable factors should be considered.

In addition to the antimicrobial effects, vitamin D is associated with modulating inflammation, hence higher levels may attenuate inflammation and more severe clinical disease [25–26, 48]. Hence, a measure of increased immunity may not be the mechanism of action by which vitamin D may function. Rather, higher vitamin D levels may reduce the severity of the inflammatory host response brought on by certain infections and inflammatory conditions. Epidemiological studies have demonstrated a connection between 25(OH)D and respiratory infections, to include viral infections, tuberculosis, and pneumonia [14, 17, 19]. Also lower levels of 25(OH)D are more frequently noted during winter months, which correlate with the seasonality of influenza. In addition to the inverse relationship between 25(OH)D levels and respiratory infections, vitamin D has been associated with IgE sensitization, which is associated with allergic inflammation [14, 21, 49–51]. However, in our study, there were no significant differences between 25(OH)D levels and ILIs, respiratory encounters, or any medical diagnoses that were analyzed.

Limitations of our study include variability in time between pH1N1 vaccine receipt and 25(OH)D measurement, therefore, associating vaccine response to a subsequent vitamin D measurement may not answer the question of how 25(OH)D levels prior to or at the time of vaccination may affect immune response to the vaccine. With our study utilizing pre-existing serum and data, we were unable to collect 25(OH)D levels at time of vaccination, nor could we check pre and post titers after vaccination. However, we adjusted our models for vaccine timing in relationship to the blood sampling and also evaluated a time period post vaccination when seroprotective antibodies would be expected. Pre-pandemic seroprevalence has been shown up to 12.5% of young adult subjects in one large German study [52]; therefore, prior exposures to the pH1N1 are not full characterized and could not be measured in this study. There are multiple factors that impact influenza rates and ILIs, however as we utilized a large, random sample, we anticipate that these would be balanced over the entire group. Since we evaluated immune responses using sera, we were not able to include T cell studies to evaluate lymphocyte function or other innate factors against influenza. The clinical outcomes in our study were based on ICD-9 codes requiring a medical visit, therefore we did not capture outcomes for which the member did not seek care or had a milder clinical course. ICD-9 codes are physician diagnosed conditions, so inaccuracies in coding may occur and influence the results. Other variables of interest, such as tobacco use, BMI, vitamin D supplement or intake and persons with higher risks for exposures (healthcare workers, other occupational or daycare exposure) were not available for analysis. We chose serum from February 2010, which would likely represent the nadir of 25(OH)D levels in most individuals. Therefore, our results, particularly the 25(OH)D levels may reflect a specific time of year, however, all studies are subject to this constantly changing biomarker. Our study population was a young military population in the U.S., and therefore may not be representative of other populations. Finally, our study is observational rather than a prospective-controlled trial, so there may be factors that impact influenza rates, vaccination patterns, and medical encounters that may affect the findings.

Our study strengths including a large sample size for this type of study, equal sampling of men and women, and uniform and concurrent testing of all samples by the same laboratory. In addition, we collected clinical data for relevant healthcare outcomes for up to 365 days after vaccination, a novel aspect of the current study.

## Conclusions

In summary, there was no relationship between 25(OH)D levels and post-vaccine antibody levels in a healthy young adult population. Vitamin D insufficiency/deficiency was highly prevalent in our population and this group had poor vaccine antibody responses and/or durability, however, 25(OH)D levels did not explain the poor post-vaccination responses or ILIs. Influenza continues to be a significant annual seasonal epidemic, therefore continued efforts identifying modifiable host factors to improve host immunity is crucial. Further research is needed to investigate host factors that may enhance the protective efficacy and durability of influenza vaccinations.

## Supporting information

S1 File Vitamin D raw data file. (XLSX)

S2 File Vitamin D microneutralization and analysis file. (XLSX)
